# Data reconstruction using iteratively reweighted L1-principal component analysis for an electronic nose system

**DOI:** 10.1371/journal.pone.0200605

**Published:** 2018-07-25

**Authors:** Hong-Min Jeon, Je-Yeol Lee, Gu-Min Jeong, Sang-Il Choi

**Affiliations:** 1 Department of Data Science, Dankook University, 152, Jukjeon-ro, Suji-gu, Yongin-si, Gyeonggi-do, 16890, Korea; 2 Department of Computer Science and Engineering, Dankook University, 152, Jukjeon-ro, Suji-gu, Yongin-si, Gyeonggi-do, 16890, Korea; 3 Electrical Engineering, Kookmin University, 861-1, Jeongneung-dong, Seongbuk-gu, Seoul 02707, Korea; Chongqing University, CHINA

## Abstract

We propose a method to reconstruct damaged data based on statistical learning during data acquisition. In the process of measuring the data using a sensor, the damage of the data caused by the defect of the sensor or the environmental factor greatly degrades the performance of data classification. Instead of the traditional PCA based on L2-norm, the PCA features were extracted based on L1-norm and updated by iteratively reweighted fitting using the generalized objective function to obtain robust features for the outlier data. The damaged data samples were reconstructed using weighted linear combination using these features and the projection vectors of L1-norm based PCA. The experimental results on various types of volatile organic compounds (VOCs) data show that the proposed method can be used to reconstruct the damaged data to the original form of the undamaged data and to prevent degradation of classification performance due to data corruption through data reconstruction.

## Introduction

The human olfactory sense is easily fatigued and cannot sustain smell; it also has a limitation whereby it cannot always precisely distinguish between similar smells. In contrast, an electronic nose system can continuously collect gas data and easily distinguish gas types, which is an advantage in various fields in which the human nose cannot be utilized [1, 2].

The electronic nose system that classifies the types of gas can be roughly divided into a sensor part that measures gas data and a computing system that extracts the features of the gas from the measured data and identifies the type of gas through the classifier [3, 4]. Sensors commonly used in electronic nose systems are electrochemical sensors such as a metal-oxide sensor [5], tin-oxide sensor [6], and piezoelectric sensor such as a carbon-black senor [7] or a conducting organic sensor [8].

The computing system consists of three steps: a preprocessing step involving converting the measured data into a form suitable for feature extraction, a step involving extracting features for gas classification, and a step involving a classifier for identifying the type of gas with the extracted features. The features for gas classification can be extracted based on various statistical methodologies widely used in the field of pattern recognition [9–11]. Various methods based on the linear discriminant analysis (LDA) [9, 12–14] or the principal component analysis (PCA) [10, 15, 16] can be used for efficient classification of high-dimensional data such as electronic nose data.

In most studies on feature extraction, it is assumed that the used data has no defect, so that if the data is partially lost or damaged, the intended performance cannot be obtained. However, in the case of the electronic nose system, since the system operating environment in the practical field is often poor, it may be difficult to collect high-quality data due to problems such as power supply or sensor defect. In this case, the classification performance of the probe data may be significantly degraded as it differs from the data used as the training data of the feature extraction.

To solve this problem, statistical analysis methods can be used to restore corrupted data and the reconstructed data can then be used for classification. In [11, 17], a conventional PCA based on L2-norm was used for data reconstruction. However, the L2-norm based PCA finds feature values to minimize the squared error between the sample and the reconstructed sample, which can excessively increase the sensitivity of the outliers [18]. Also, since the PCA features are values obtained from a linear transformation of the data samples by the projection vectors, if noise or defects occur in the training data, distortion occurs in the projection vectors, rendering it difficult to obtain good features. In [19], joint formulation of recovering low-rank and sparse subspace structures was proposed for robust representation and classification. In [20, 21], the discriminative feature extraction method, which integrates linear subspace learning and low-rank matrix recovery, was proposed to improve classification performance. The method in [22] extracted discriminative features using the data from multiple views for times series classification.

In this paper, we propose a method to reconstruct a data sample, some values of which are lost due to sensor instability in the electronic nose system. The proposed method is composed of a part for obtaining a feature vector for representing data in a low dimensional space and a part for updating the feature values appropriately for data restoration. First, by using L1-norm maximization-based PCA (L1-PCA) [18], projection vectors less affected by outlier samples are obtained and the initial features are obtained through a linear transformation of data samples using projection vectors. Then, by repeatedly updating the initial feature values to satisfy the generalized objective function for the errors between the reconstructed sample and the original sample [23], better features for use in data reconstruction were obtained. While L1-PCA is performed in the training phase, only the update of the feature values for the distorted sample is performed in the test phase. The gas data samples reconstructed using the updated new features are classified through the discriminant feature extraction process and the classifier. The main contribution of this paper is as follows. 1) As a variant of PCA, we proposed a more specialized method for data reconstruction. 2) By applying the proposed data reconstruction method to the electronic nose data, the performance of the gas classification is improved, by alleviating the influence of the damage that occurs in the data acquisition process using the sensor.

For the reconstruction experiment of lost data, we used data measurement using the carbon-black sensors for 8 types of gas, and we partially lost values of the data randomly [24]. We then evaluated the reconstruction performance by measuring the root mean squared (RMS) error of the reconstructed result using the proposed method and the lossless data. In addition, we confirmed the way in which the proposed reconstruction process can improve the gas classification performance by comparing the classification rates before and after reconstruction.

This paper is structured as follows. In the next section, we present the data reconstruction method using iteratively reweighted L1-principal component analysis. Then, we design the electronic nose system using the proposed data reconstruction method. Finally, the experimental results on data reconstruction and gas classification are described and the conclusion follows.

## Data reconstruction using iteratively reweighted L1-principal component analysis

### Iteratively reweighted L1-PCA

When dealing with high-dimensional data such as electronic nose data, we can simplify the problem for effective analysis by using the dimension reduction method. PCA, which is a multi-variate analysis method based on statistical methodology, is one of the most popular methods for this purpose.

Let us consider a data set consisting of *N* samples. Each sample can be represented by a point **x**_*k*_ = [*x*_*k*1_, ‥, *x*_*kn*_]^*T*^ in the *n*-dimensional vector space. This space is called an input space, and each component of **x**_*k*_ is called a primitive variable. In the conventional PCA, we find the projection vectors **w**_*l*_ = [*w*_*l*1_, ‥, *w*_*ln*_]^*T*^, *l* = 1, …, *m* that satisfy the following objective function based on L2-norm [25].

$$\begin{array}{c} J_{m}=\sum_{k=1}^{N}\left|\right|\left(\mu +\sum_{l=1}^{m}y_{kl}w_{l}\right)-x_{k}{\left|\right|}^{2} \end{array}$$ (1)

Here, *μ* is the sample mean $\mu =\frac{1}{N}\sum_{k=1}^{N}x_{k}$ and *y*_*kl*_s are principal components (PCA features corresponding to **w**_*kl*_s).

The global minimum of *J*_*m*_ can be obtained by using the singular value decomposition (SVD) [26] to find *W* that satisfies the following object function.

$$\begin{array}{c} W\left(=\left[w_{1},w_{2},‥,w_{m}\right]\right)=\text{arg}\underset{W}{\text{max}}\left|\right|W^{T}S_{T}W\left|\right| \end{array}$$ (2)

Here, *S*_*T*_ is a total scatter matrix and is defined as $S_{T}=\sum_{k=1}^{N}\left(x_{k}-\mu \right){\left(x_{k}-\mu \right)}^{T}$.

However, since the conventional PCA constructs a feature space that maximizes the dispersion of the samples based on the L2-norm, when an outlier data sample is present, the sample tends to have an excessive influence on the process of obtaining the projection vector. Therefore, we use L1-PCA [18] based on L1-norm, which is more robust to outlier data than L2-norm for data reconstruction. In order to prevent distortion of the equidistance surface by the rotation of L1-norm, L1-PCA finds a projection vector that maximizes the L1 dispersion using L1-norm in the feature space by using the following objective function.

$$\begin{array}{ccc} W^{*}=\text{arg}\underset{W}{\text{max}}\sum_{k=1}^{N}\sum_{l=1}^{m}\left|\sum_{i=1}^{n}w_{li}x_{ki}\right| & & \\ \text{subject}\;\text{to}\;W^{T}W=I\in R^{m\times m} & \end{array}$$ (3)

The optimal *l*-th projection vector, **w**_*l*_, satisfying the objective function in (3) is changed according to the number of projection vectors (*m*) to be obtained and it is very difficult to obtain the global solution for (3) when *m* > 1. In order to avoid this problem, as in [18], we also obtain w* by using the following objective function when *m* = 1.

$$\begin{array}{c} w^{*}=\text{arg}\underset{W}{\text{max}}\sum_{k=1}^{N}|w^{T}x_{k}{\left|\;\text{subject}\;\text{to}\;||w|\right|}_{2}=1 \end{array}$$ (4)

Then, we find an approximate solution (*W*_*L*1_ = [**w**_1_, **w**_2_, ‥, **w**_*m*_]) to (3) by using the greedy search method [18].

By using *W*_*L*1_, the feature vector for the data sample is obtained through the following linear transformation.

$$\begin{array}{c} y_{k}=W_{L1}^{T}\left(x_{k}-\mu \right) \end{array}$$ (5)

The features are then updated through the iteratively reweighted fitting (IRF) process [23] to improve the effect of data reconstruction. To achieve this, a generalized objective function containing nonlinear mapping is defined as (6) and the projection vector is repeatedly weighted using the iteratively reweighted least squares (IRLS) [27].

$$\begin{array}{ccc} J(y) & = & \sum_{i=1}^{n}G\left({\left(x_{i}-W_{i}y\right)}^{2}\right)\\ \text{G}(z) & = & \log\frac{1}{1+\exp\left(-\beta \left(z-\eta \right)\right)} \end{array}$$ (6)

In (6), *β* and *η* (which are tuning parameters) are the inverse temperature and saturation value, respectively, and *W*_*i*_ denotes the *i*-th row vector of *W*_*L*1_.

The process of minimizing the objective function in (6) can be divided into a weight calculation step and a least squares step [23]. In the weighting step at each (*t*-th) iteration, a weight vector $\omega^{\left(t\right)}={\left[\omega_{1}^{\left(t\right)},\omega_{2}^{\left(t\right)},‥,\omega_{n}^{\left(t\right)}\right]}^{T}$ is defined for a feature vector **y**^(*t*)^, and its values are calculated as follows [23].

$$\begin{array}{c} \omega_{i}^{\left(t\right)}=\frac{\exp\left(-\beta \left(z_{i}^{\left(t\right)}-\eta \right)\right)}{1+\exp\left(-\beta \left(z_{i}^{\left(t\right)}-\eta \right)\right)} \end{array}$$ (7)

$$\begin{array}{c} z_{i}^{\left(t\right)}={\left(x_{i}-W_{i}y^{\left(t\right)}\right)}^{2} \end{array}$$ (8)

In the least squares step at the (*t* + 1)-th iteration, the feature vector **y**^(*t*+1)^ is updated with the weight vector ***ω***^(*t*)^ calculated in the weight step as follows.

$$\begin{array}{c} y^{\left(t+1\right)}={\left(\sum_{i=1}^{n}\omega_{i}^{\left(t\right)}W_{i}^{T}W_{i}\right)}^{-1}\sum_{i=1}^{n}\omega_{i}^{\left(t\right)}W_{i}^{T}x_{i} \end{array}$$ (9)

In this manner, while repeating the weighting step and the least square step, the feature vector (**y**^(*t*)^) updating is repeated until the convergence or termination condition (*t* = *t*_*max*_) is satisfied.

### Reconstruction of distorted data

**Fig 1:**
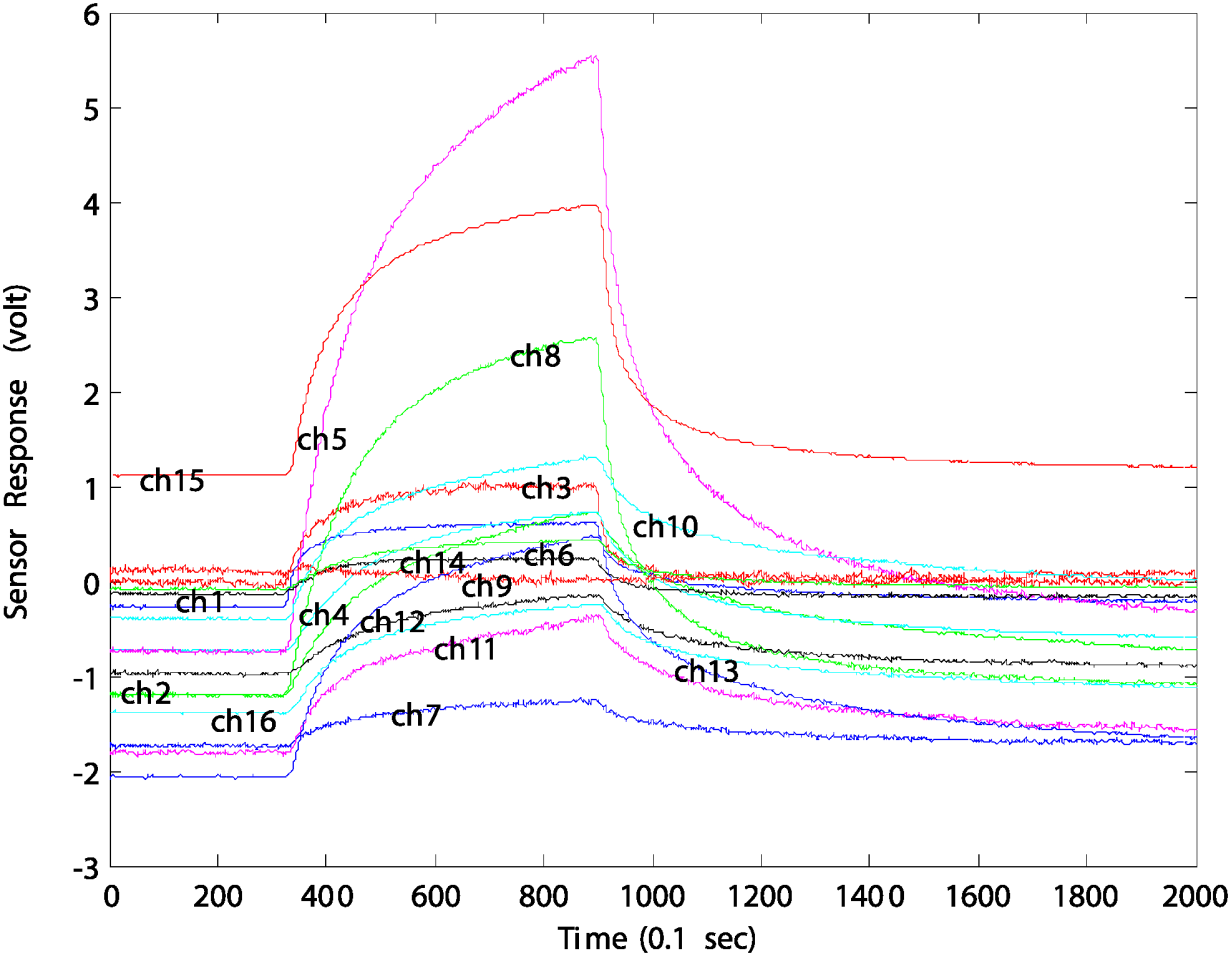
Typical time-response of 16 channel sensor array with respect to inflow of ethanol vapor.

Fig 1 shows typical time-responses of a 16 channel sensor array for ethanol vapor. In the case of sensor data, data measurement may be partially lost or damaged depending on the installation environment and electrical environmental conditions. The lost or damaged data can be reconstructed using the projection vectors of the L1-PCA and the updated new L1-PCA features, which can be accomplished by simple matrix operations.

As shown in (3), the projection vectors obtained through L1-PCA are orthogonal to each other; hence, **x**_*k*_ is approximated as a linear combination of the basis **w**_*l*_s that constitutes a feature space as follows.

$$\begin{array}{c} x_{k}=W_{L1}y_{k}+\mu \end{array}$$ (10)

The reconstructed data **x**^*re*^ for the damaged data sample **x**^*dmg*^ can be obtained by using *m* projection vectors with high data representation power and the feature vector $y^{\left(t\right)}={\left[y_{1}^{\left(t\right)},y_{2}^{\left(t\right)},‥,y_{m}^{\left(t\right)}\right]}^{T}$ updated through the IRF as follows.

$$\begin{array}{ccc} y_{k} & = & W_{L1}^{T}\left(x^{dmg}-\mu \right)\underset{IRF}{⟶}y_{k}^{\left(t\right)}\\ x_{k}^{re} & = & W_{L1}y_{k}^{\left(t\right)}+\mu \end{array}$$ (11)

**Fig 2:**
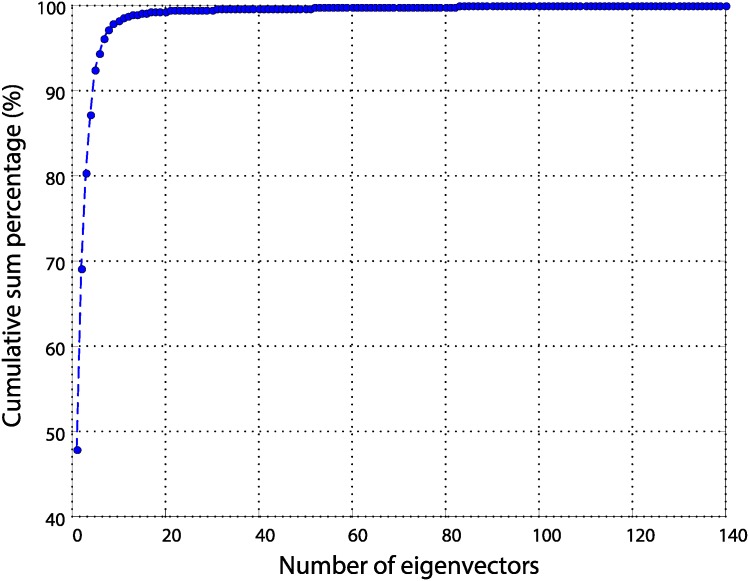
Cumulative sum percentage of eigenvalues after sorting the eigenvalues of the scatter matrix of e-nose data samples in descending order.

Fig 2 shows a graph plotting the cumulative sum percentage of eigenvalues after sorting the eigenvalues of the scatter matrix of electronic nose data samples in descending order. In Fig 2, the magnitude of the eigenvalue *λ*_*l*_ decreases sharply at the beginning with an increasing index *l*, which means that most of the eigenvalues are concentrated in a few major eigenvectors. The eigenvalue of the projection vector refers to the variance of the data samples in the feature space. However, the estimated eigenvalue *λ*_*l*_ from the training samples somewhat differs from the true variance of the projected vector, due to the limited number of training samples. In particular, eigenvectors with small eigenvalues are sensitive to noise [28]. Therefore, in this paper, we only use eigenvectors with large eigenvalues instead of using whole eigenvectors in the data reconstruction process.

In order to determine the optimal *m* value for data reconstruction, the root mean squared (RMS) error between the data before loss and the reconstructed data defined as in (12) was calculated.

$$\begin{array}{c} {\text{E}}_{RMS}=\frac{1}{N}\sum_{k=1}^{N}\left|\right|x_{k}-x_{k}^{re}{\left|\right|}_{2} \end{array}$$ (12)

**Fig 3:**
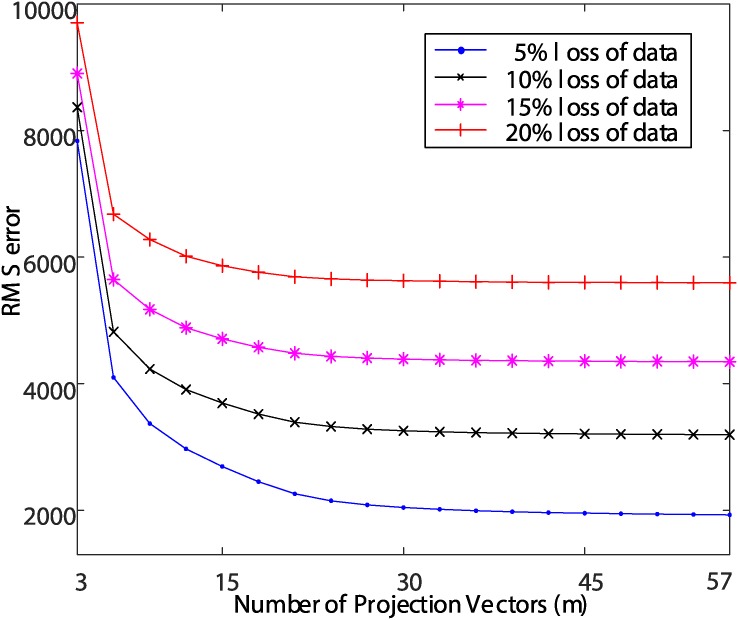
Observations of RMS errors for different numbers of projections vectors (*m*).

Fig 3 shows the RMS errors when the data is reconstructed using the *W*_*L*1_ composed of *m* L1-PCA projection vectors and the feature vector **y**^(*t*)^ while varying the value of *m*, given a loss of an arbitrary ratio to the values of the training data samples. Fig 3 shows that when the number of eigenvectors used for reconstruction is small, the RMS error decreases sharply as the number of eigenvectors increases, and RMS errors converge when a certain degree of eigenvectors are secured. This can be seen in the same context as the interpretation of Fig 2 mentioned above. In this paper, we evaluate the reconstruction performance by changing the *m* value several times based on the result of Fig 3, and set the value of *m* to 15.

**Fig 4:**
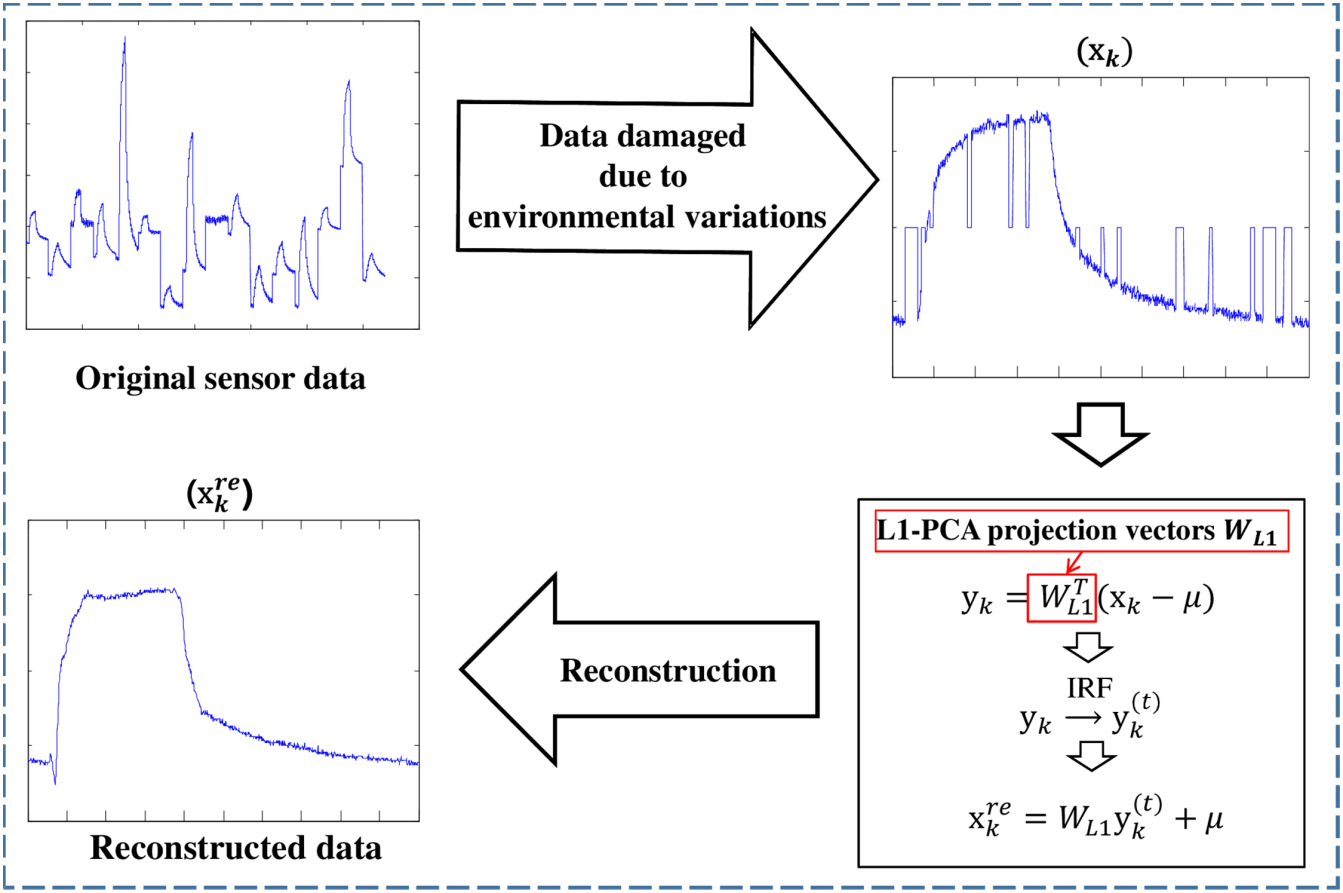
Overall procedure of the proposed data reconstruction method.

Fig 4 shows the overall procedure of the proposed data reconstruction method using the iterative reweighted L1-PCA.

## Design of electronic nose system

### Data acquisition

**Fig 5:**
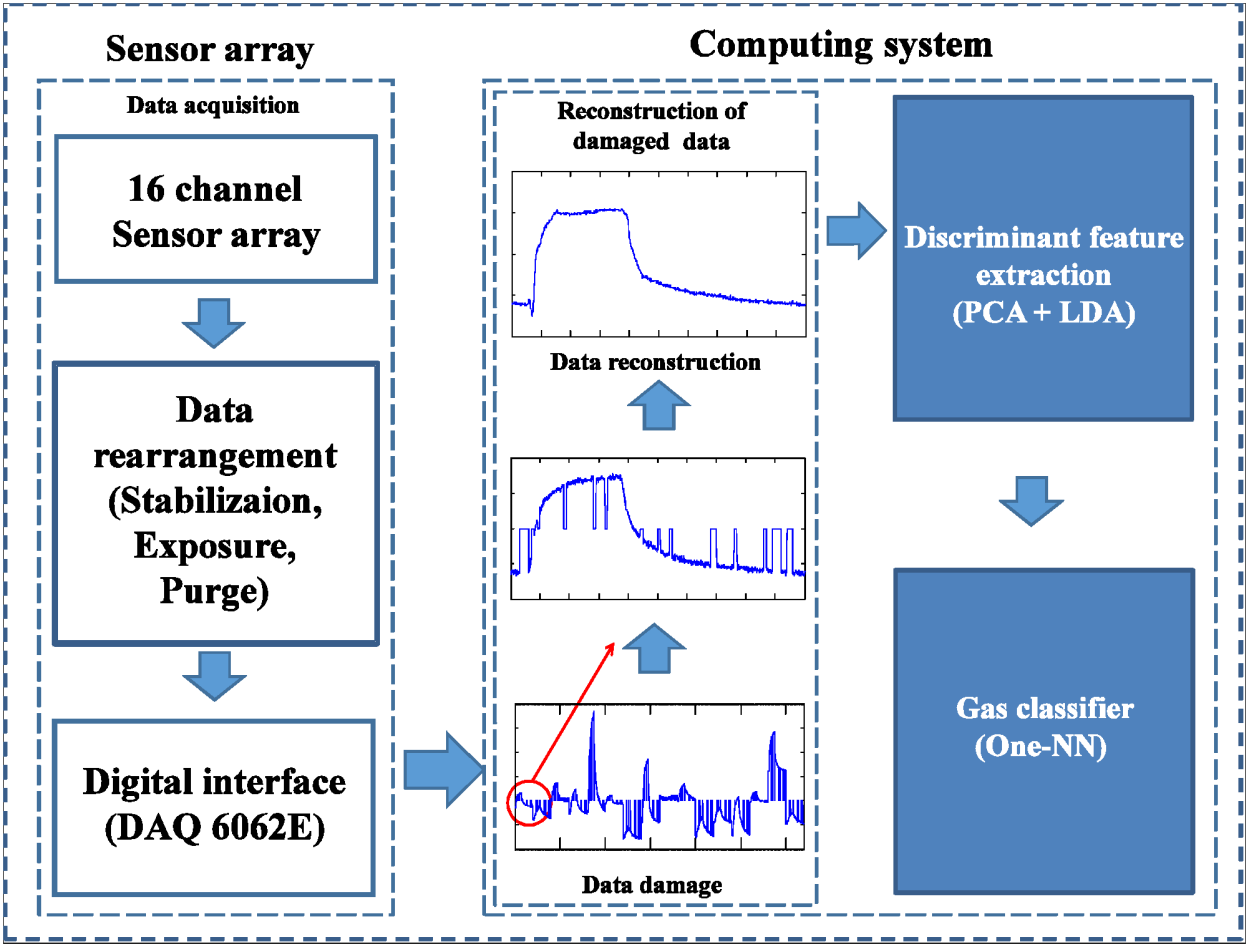
Schematic diagram of our electronic nose system. The gas data sample is stored as a vector through a digital interface. Then, the computing system classifies the types of gas through the data reconstruction and feature extraction steps.

Fig 5 shows a schematic diagram of the electronic nose system used in this paper. While polymer composites have limitations in sensor life, sensor drift, and sensitivity to temperature and humidity, they are widely used in electronic nose systems compared to other gas sensors due to low cost, low power, stable operation at room temperature, etc. [11, 16, 29].

In the electronic nose system used in this paper, a micoromachined sensor array chip used in [11] was used. The sensor array consists of 16 channels, and each channel has a carbon-black (CB) polymer composites sensor with an interdigitated electrode, a microheater, and a machined membrane. Table 1 shows 16 types of (CB) polymer composites. The measurement of the sensor was performed by observing the change in resistance when the chemical gas was bonded to each polymer composite film and recording it for a total of 200 seconds at 0.1 second intervals.

**Table 1:** CB polymer composites in the sensor array.

Channel	Polymer
1	Poly(methyl methacrylate)
2	Polyvinylpyrrolidone
3	Poly(vinyl acetate)
4	Poly(ethylene oxide)
5	Polycaprolactone
6	Poly(4-methylstyrene)
7	Poly(styrene-co-methyl methacrylate)
8	Poly(enthylene-co-vinylacetate)
9	Poly(bisphenol A carbonate)
10	Poly(4-vinyl pyridine)
11	Poly(vinyl butyral)-co-vinyl alcohol-co-vinyl acetate
12	Poly(vinyl stearate)
13	Ethyl cellulose
14	Polystyrene-block-polyisoprene-block-polystyrene
15	Hydroxypropyl cellulose
16	Cellulose acetate

First, after the sensor array is placed in the chamber and the resistance signal stabilizes for 30 seconds (stabilization), the flow control of the system exposes the gas for 60 seconds (exposure) and leaves the remaining gas to the outside for 110 seconds [27]. The measured data are stored on a PC using the DAQ6062E data acquisition (DAQ) board and LabVIEW (National Instrumentation, USA). The voltage-divider operates from -10V to 10V and the gain of 16 identical amplifiers is set to 10 for maximum DAQ resolution [24].

### Feature extraction for classification from reconstructed data

If extracting features that are effective for gas classification from the reconstructed data, the classifier takes these features as inputs and finally determines the type of gas. In this paper, we use the linear discriminant analysis (LDA) method [10], which is a typical supervised learning method, as a feature extraction method for classification. LDA constructs a low-dimensional feature space such that the ratio of the variance of each class mean and the variance of the samples in the same class increases. While feature extraction methods other than LDA can also be employed for this purpose, the LDA method was selected in this study for convenience.

When *N* training data samples **x**_*k*_ (*k* = 1, …, *N*) are composed of *C* classes and each class *c*_*i*_ (*i* = 1, …, *C*) has *N*_*i*_ samples, the between-class scatter matrix (*S*_*B*_) and the within-class scatter matrix (*S*_*W*_) are defined as follows.

$$\begin{array}{ccc} {\text{S}}_{B} & = & \frac{1}{N}\sum_{i=1}^{C}N_{i}\left(\mu_{i}-\mu \right){\left(\mu_{i}-\mu \right)}^{T}\\ S_{W} & = & \sum_{i=1}^{C}\sum_{x_{k}\in c_{i}}^{}\left(x_{k}-\mu_{i}\right){\left(x_{k}-\mu_{i}\right)}^{T}\\ \mu_{i} & = & \frac{1}{N_{i}}\sum_{x_{k}\in c_{i}}^{}x_{k},\;\mu =\frac{1}{N}\sum_{i=1}^{C}\sum_{x_{k}\in c_{i}}^{}x_{k} \end{array}$$ (13)

LDA constitutes a feature space that can be distinguished between classes by maximizing the ratio of *S*_*B*_ and *S*_*W*_. Therefore, the objective function of LDA can be expressed as follows.

$$\begin{array}{c} {\text{W}}_{LDA}=\text{arg}\underset{W}{\text{max}}\frac{|W^{T}S_{B}W|}{|W^{T}S_{W}W|} \end{array}$$ (14)

The solution satisfying (14) corresponds to the eigenvector of $S_{W}^{-1}S_{B}$. In the high-dimensional data such as the electronic nose sensor data, the small sample size (SSS) problem [30] occurs in which the number of training data is smaller than the dimension of training data, and no inverse matrix is available. To avoid this problem, we first reduce the dimension of data to less than the rank of *S*_*W*_ using PCA and then applied LDA in the PCA feature space (PCA + LDA [9]). If letting the projection matrix of the PCA be *W*_*PCA*_, the final projection matrix by PCA + LDA can be expressed as follows.

$$\begin{array}{ccc} {\text{W}}_{PCA+LDA}=W_{LDA}^{T}W_{PCA}^{T} & & \\ W_{LDA}=\text{arg}\underset{W}{\text{max}}\frac{|W^{T}W_{PCA}^{T}S_{B}W_{PCA}W|}{|W^{T}W_{PCA}^{T}S_{W}W_{PCA}W|} & \end{array}$$ (15)

If selecting *n*′(≤*C* − 1) projection vectors constituting *W*_*PCA*+*LDA*_ in order of their eigenvalues, the gas data sample **x**_*k*_ is an *n*′-dimensional feature vector composed of *n*′ discriminant features as follows.

$$\begin{array}{c} y_{k}^{L}=W_{PCA+LDA}^{T}\left(x_{k}-\mu \right)={\left[y_{k1}^{L},y_{k2}^{L},‥,y_{kn^{\prime }}^{L}\right]}^{T} \end{array}$$ (16)

## Experimental results

### Reconstruction of electronic nose data

**Fig 6:**
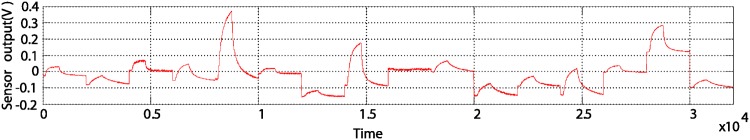
Representation of data sample in 32,000 dimensional vector form.

In order to verify the effectiveness of the proposed method, we attempted to classify the volatile organic compounds (VOCs) measurement data for 8 types of gases. The gases used in the experiments were acetone, benzene, cyclo-hexane, ethanol, heptane, methanol, propanol, and toluene [24]. Twenty samples were collected for each type of gas and a total of 160 samples were collected. Each sample consists of the measurements for 2,000 time points measured at a sampling rate of 10 Hz per channel for 200 seconds. The measurement values of 16 channels are stored in the form of 2,000 × 16 matrix, and then converted to a 32,000 dimensional vector using a lexicographic ordering operator [29] (Fig 6).

To see the effectiveness of the proposed method in reconstructing the data, we analyzed the performance for the data samples with data loss of 20% ($x_{20\%}^{dmg}$) of the total measurements and the data sample ($x_{GN}^{dmg}$) to which the random Gaussian noises were added. For this purpose, considering the electrical problems that may occur in the actual electronic nose installation environment, it is assumed that the loss interval occurs in 2 second units (20 time points), and the data value of the corresponding interval is set to zero. All data values used in the experiments were normalized [29] using the mean and standard deviation of the training data.

**Fig 7:**
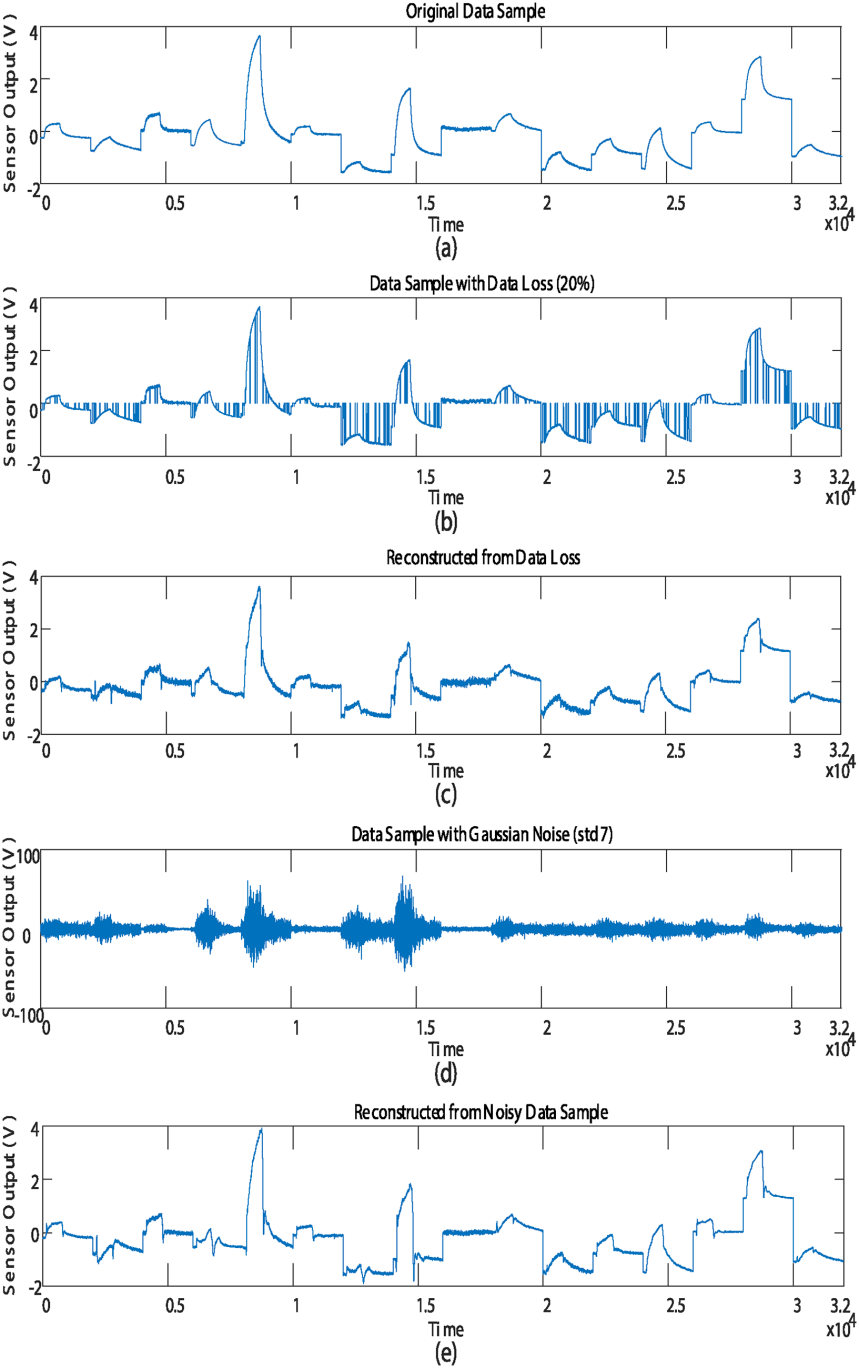
Representation of the data sample before and after the reconstruction process. (a) Original data sample. (b) Data samples with data loss $x_{20\%}^{dmg}$. (c) Reconstructed data sample from loss $x_{20\%}^{re}$. (d) Data sample with Gaussian noise $x_{GN}^{dmg}$. (e) Reconstructed data samples from noise $x_{GN}^{re}$.

Fig 7 shows (a) the data samples having the loss ($x_{25\%}^{dmg}$) and (b) the data sample with Gaussian noise ($x_{GN}^{dmg}$). As shown in Fig 7, the shapes of the damaged data samples were reconstructed by the proposed method to be similar to the respective shapes of the original data.

For a total of 160 gas data samples of 8 types, the gas classification experiments were conducted to verify the effect of the data reconstruction on the gas classification performance of the electronic nose. All samples were tested using 8-fold cross validation [31]. In other words, data were randomly mixed and then divided into training data sets consisting of 140 samples and test data of 20 samples for each fold. The final classification rates were calculated by averaging the classification rates in 8 experiments.

As mentioned previously, the discriminant features to be used as input to the classifier were extracted using the PCA + LDA method. In the PCA phase of PCA+LDA, the dimension of original sample space (32,000 dim.) was reduced to the 105 dimensional feature space corresponding to 99% of the total eigenvalues of *S*_*T*_, and then, LDA was performed in the reduced feature space. Since the PCA+LDA method can extract up to 7 features in the problem of 8 classes, the classification performance is measured in the 7-dimensional PCA + LDA feature space. The feature vector for **x**^*dmg*^ and **x**^*re*^ can be expressed in a maximum 7-dimensional space as **y**^*dmg*^ and **y**^*re*^. One-nearest neighbor (One-NN) classifier was used as the classifier, and the distance between samples was measured based on L2-norm [11]. Similar to the reason for using PCA + LDA, we used One-NN using L2-norm based distance measure for convenience. The time required for the L1-PCA to obtain the projection vectors is about 0.20s, which is slightly longer than that of L2-PCA (about 0.15s), but this is done only in the training process.

**Fig 8:**
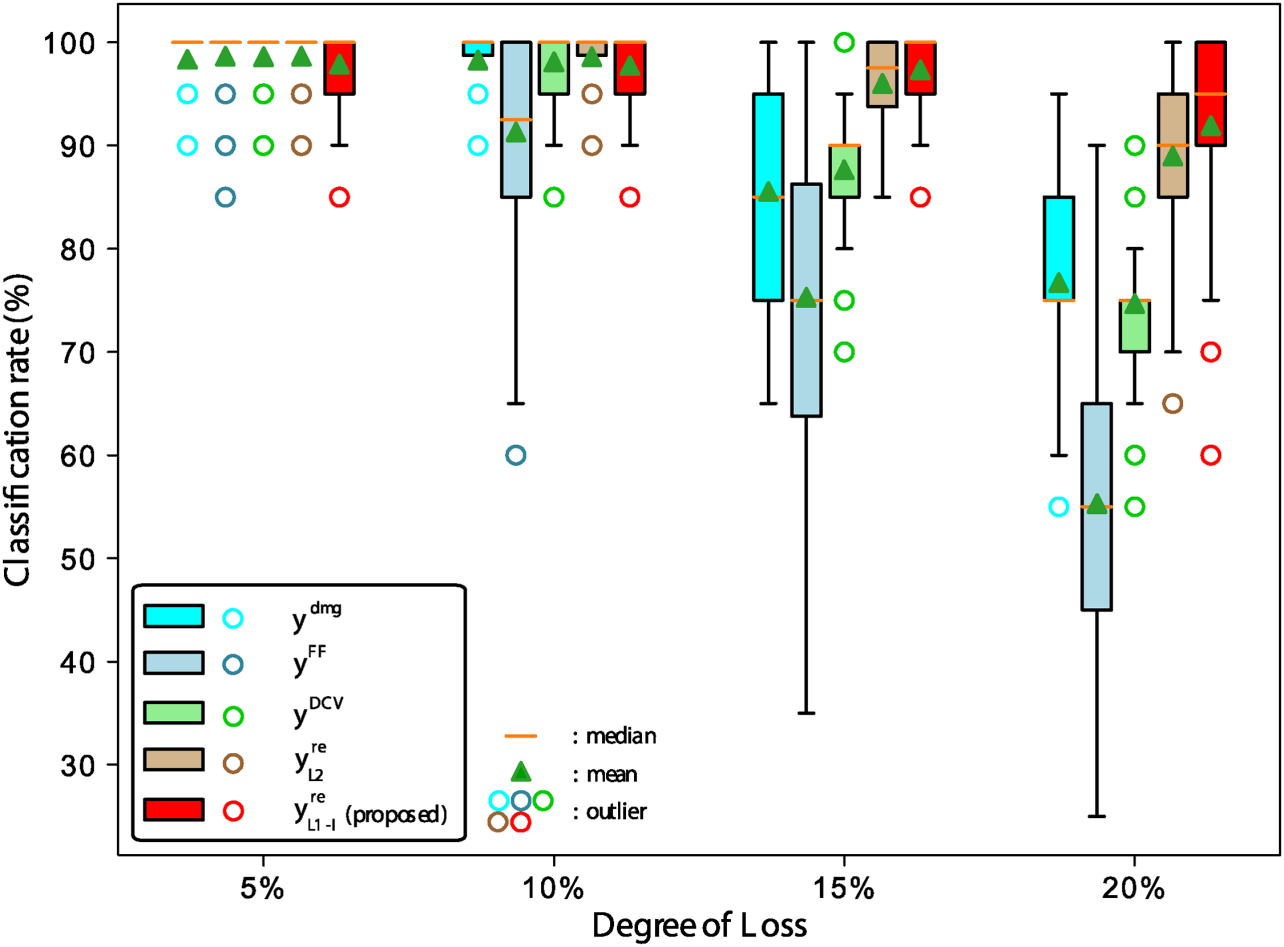
Comparison of classification rates between the proposed method and other methods.

**Fig 9:**
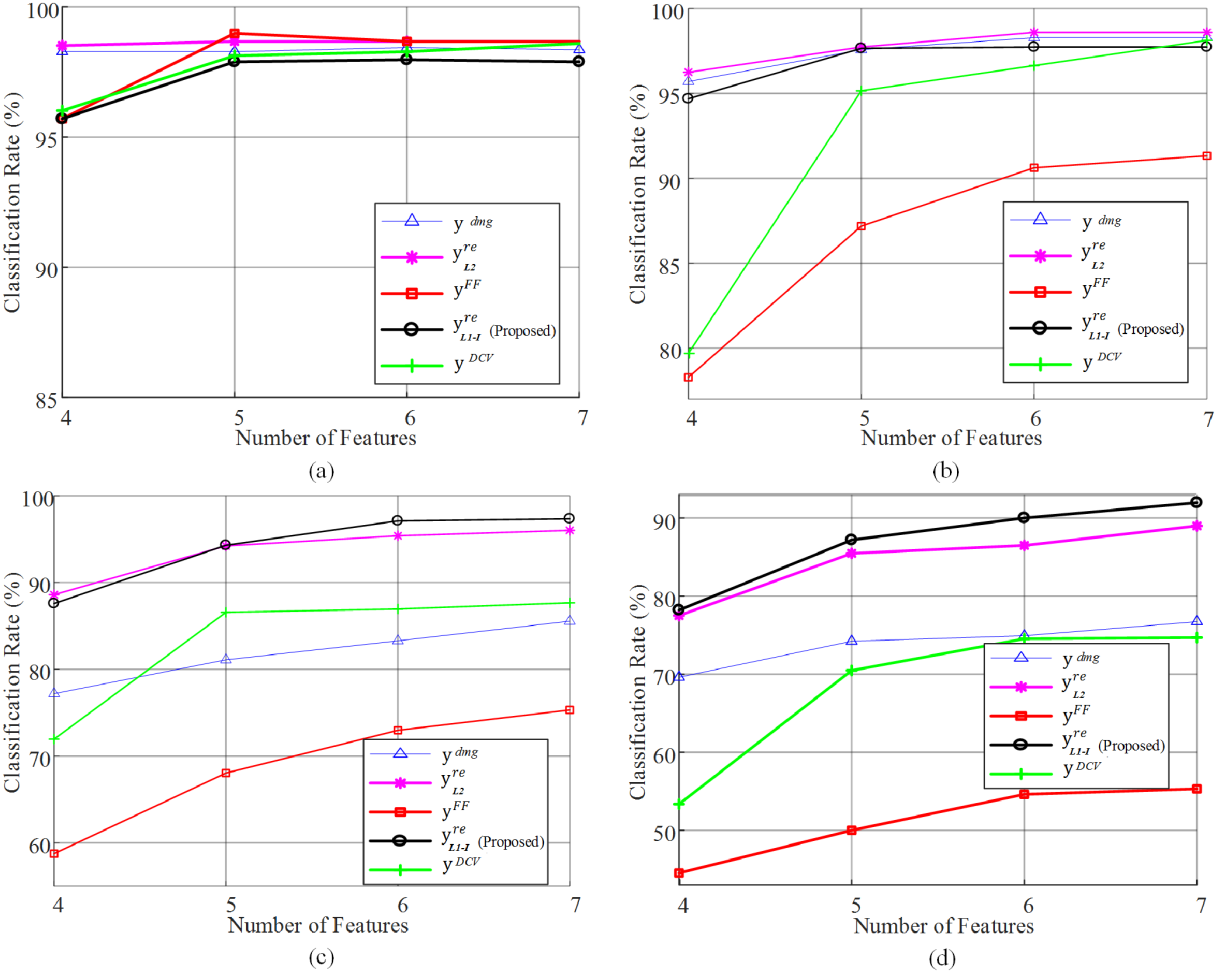
Classification rates for various dimensions of the feature space. (a) 5% data loss (b) 10% data loss (c) 15% data loss (d) 20% data loss.

We compared the classification performance of the proposed method $\left(y_{L1-I}^{re}\right)$ with that of other methods for electronic nose classification, including FF (Feature Feedback) method (**y**^*FF*^) [32], the DCV (Discriminant Common Vector) method (**y**^*DCV*^) [14], and the L2-PCA based data reconstruction method $\left(y_{L2}^{re}\right)$ [17]. Classification rates were obtained from data samples of the loss of 5% ∼ 20%. Fig 8 shows the comparison of classification rates between the proposed method and other methods and Fig 9 shows the classification rates for various dimensions of the feature space. As shown in Figs 8 and 9, each method exhibited favorable classification performance with the data sample of less loss (5% and 10%), showing that even when the data was not reconstructed ($y_{5\%}^{dmg}$ and $y_{10\%}^{dmg}$), the classification rate were as high as 98.2%. However, as the amount of data loss increased from 15% to 20%, the classification rates decreased significantly in the absence of data reconstruction. This is because the data samples that were more than 15% lost seem to have lost much of the inherent characteristics of the class in the PCA + LDA feature space. However, as the degree of data loss increases, while the classification performances of the other methods decrease rapidly, the proposed method maintains a certain level of classification performance (91.9%).

### Reconstruction of high-dimensional data - Face image

In order to confirm the effect of the proposed data reconstruction method, we experimented with face images that are high dimensional data such as electronic nose data, from the AR database [33]. The AR database contains images with many variations, such as illumination and facial expressions, and consists of two sessions taken at a two week interval. We used the images without partial occlusion for 118 subjects in the experiment. The images taken at ‘session 1’ were used as training images for image reconstruction and recognition, and the reconstruction and recognition performances were tested with neutral images in ‘session 2’.

**Fig 10:**
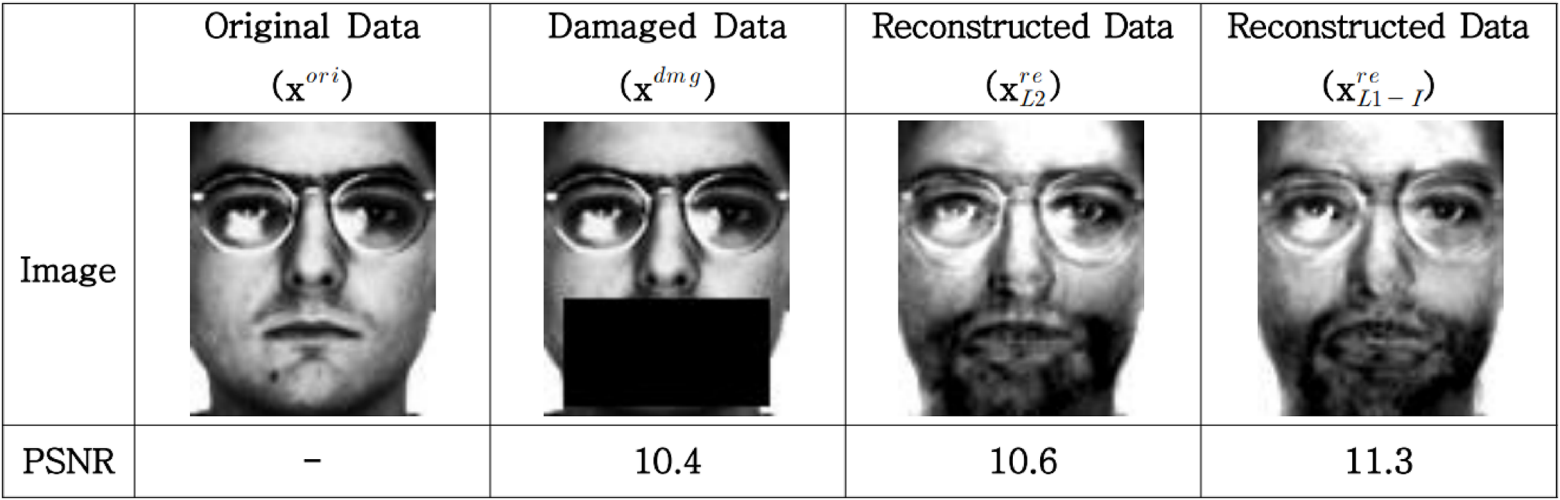
Reconstruction of an occluded face image.

Fig 10 shows the original image, the partially occluded image, and the reconstructed images using the method in [17] and the proposed method (*m* = 45). In Fig 10, the qualities of the reconstructed images by L2-PCA $\left(x_{L2}^{re}\right)$ and those of the proposed method $\left(x_{L1-I}^{re}\right)$ appear to be similar overall. However, in detail, it can be seen that the traces of the eyeglass frame in are thinner than in and the glare of the spectacle lens is effectively removed. In addition, we computed the peak signal to noise ratio (PSNR) based on the original image as $PSNR=20\cdot log_{10}\left(255/\sqrt{M}SE\right)$, where $MSE=1/N\sum_{i=1}^{N}\left|\right|x_{i}^{ori}-x_{i}^{re}{\left|\right|}^{2}$, and the PSNR of $x_{L1-I}^{re}$ is higher than that of $x_{L2}^{re}$.

**Fig 11:**
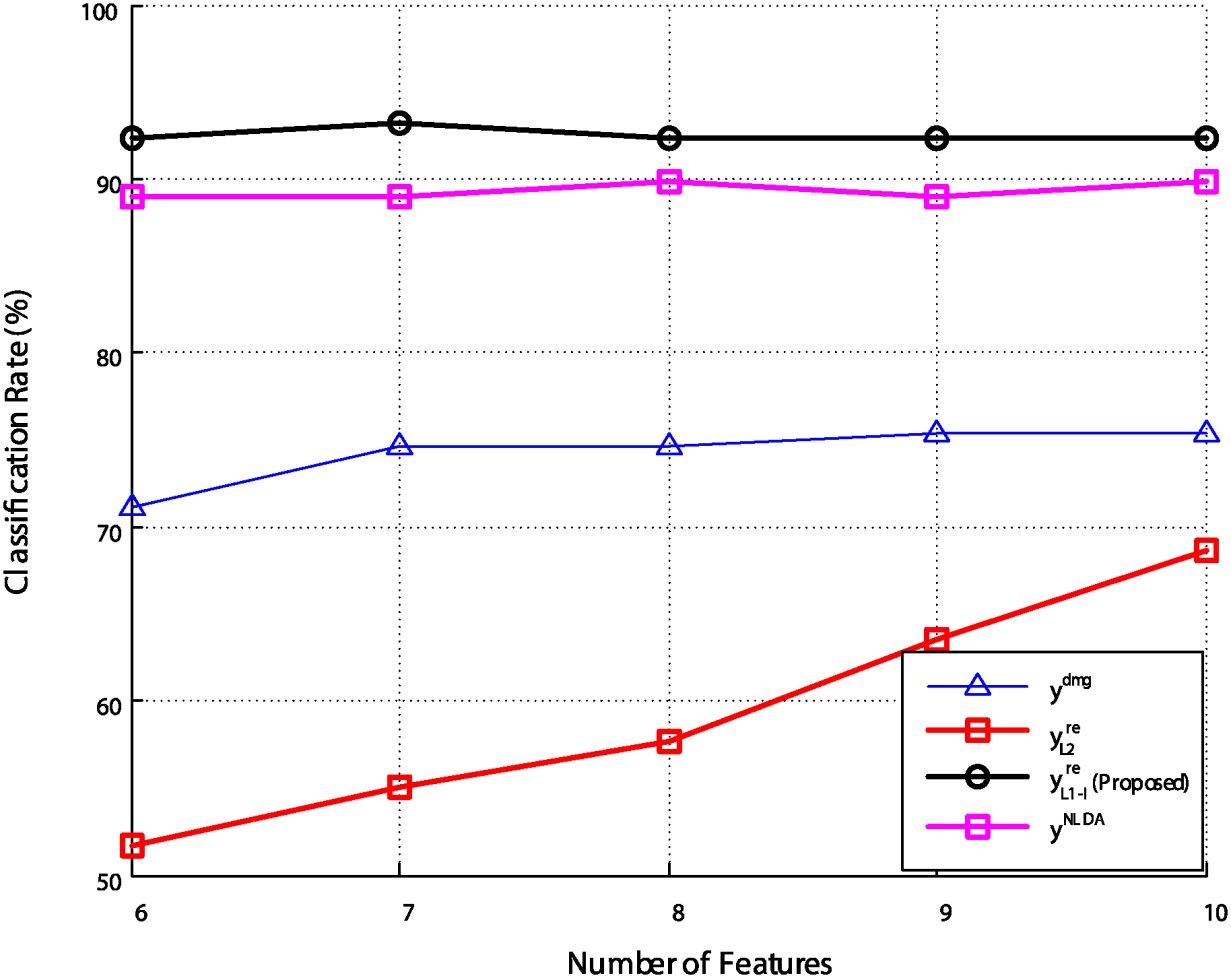
Recognition rates for various dimensions of the feature space.

We also performed recognition experiments on damaged face images and reconstructed images. As in the experiment on the electronic nose data, the discriminant features for recognition were extracted by using the PCA+LDA method, and up to 117 features were extracted. Fig 11 shows the recognition rates for various dimensions of the feature space. In Fig 11, the recognition rate of 94.1% for the original face images (**x**^*ori*^) dropped to 83.1% for the occluded face images (**x**^*dmg*^). However, by reconstructing the images by the proposed method, the recognition rate was restored to around 92.4%, which was better than the results given by the other methods.

## Conclusions

In an electronic nose system, data loss caused by the installation environment or electrical instability of the sensor deteriorates the stability of the gas classification performance. In this paper, we proposed a method to reconstruct the damaged data effectively to improve the stability of the electronic nose system. PCA is used not only for dimension reduction or representation of high-dimensional data such as electronic sensor data, but also for reconstructing the original dimension data by a linear combination of projection vectors and the PCA features. We used L1-norm based PCA, instead of conventional L2-norm based PCA, to reduce the influence of outlier data. In addition, by repeatedly updating the features using the generalized objective function for the reconstruction error, we reduced the distortion of the L1-PCA features due to the outlier samples, and obtained high-quality features. The damaged data samples were reconstructed by the weighted linear combination of the projection vectors of L1-PCA and the updated features.

In order to verify the effectiveness of the proposed method, the reconstruction and gas classification experiments were performed with eight types of gas data measured by the carbon-black sensor. As a result, the lost data was reconstructed to a shape similar to the original data. The result of the gas classification experiment on the reconstructed data confirmed that the data reconstruction process mitigates the deterioration of the gas classification performance due to the data loss.

For the implementation of a practical electronic nose system, it is important to classify the data containing combinations of gases and different concentrations, etc., while experiments need to be performed on data measured using various types of sensors. Further studies will be carried out using experiments involving various types of complex data to investigate a combination of diverse features.
